# Large Echinococcus Cyst of the Liver; Operation and Recovery1Read at the twentieth annual meeting of the American Association of Obstetricians and Gynecologists, held at Detroit, September. 17-19, 1907.

**Published:** 1908-02

**Authors:** Herman E. Hayd

**Affiliations:** Buffalo, N. Y.


### Large Echinococcus Cyst of the Liver; Operation and Recovery[1]

By HERMAN E. HAYD, M. D., M.R.C.S. Eng., Buffalo, N. Y.

[*American Journal of Obstetrics, December, 1907*]

THE tenia echinococcus is a variety of tapeworm found in the dog, and occasionally in the wolf and jackal, but very rarely in our native North American animal; in fact, only one authentic case is reported, and that by Curtice of Washington, D. C. Numerous other observers, such as Osler and Clement, Sommer, Stiles, and Hassall of the Bureau of the animal industry, have made many dissections of many different varieties of dogs, and have never once found it. It is a tiny cestode four or five millimeters in length, and consists of only three or four segments, of which the terminal one alone is mature. The head is small, and provided with four sucking disks, a rostellum, and a double row of hooklets. The ripe segment contains about 5,000 eggs. It inhabits the upper intestines, and is seen as a little, white, threadlike body, closely adherent among the villi.

To the abdominal surgeon it is of especial interest, since it produces in man in its larval stage a disease which is termed hydatids, and which is developed in various organs of the body, especially the liver, where it occurred in 73.7 per cent. of the recorded cases. It also attacks the lungs, pleura, kidneys, the muscles and dermis, the brain, the female genitalia, and the bones and eyes. In this country the disease is exceedingly rare, and when found it is usually in the foreign-born individual, but in Iceland, where dogs are used freely, and are in such intimate and constant association with the human being, the disease is so common that it is referred to as the "Liver Plague," and one in every seven or ten deaths is due to it.

---

1. Read at the twentieth annual meeting of the American Association of Obstetricians and Gynecologists, held at Detroit, September, 17-19, 1907.

In Australia it is also very common, and in certain parts of Germany, and in Winnipeg, among the Icelandic population, quite a number of cases have been found, but only one case is reported in a Canadian-born offspring. The eggs are voided in large numbers by the dog, and are deposited in water or on the various vegetables we use as food, or they may be conveyed from the body of the dog by hands to mouth. The egg is swallowed and gets into the stomach or intestines, and there its surrounding wall is digested or dissolved off, the embryo is freed and bores its way through the mucosa into a bloodvessel, and is carried to various parts of the body. Wherever it makes a connection, an inflammatory exudate is thrown out which surrounds the cell, and subsequently becomes its protecting envelope or capsule. Inside of this capsule the parasite continues to grow; it consists of two layers, an outer lamellated structure called the cuticula, and an inner granulocellular—the parenchyma; from this inner layer, the secondary or daughter cysts develop, and from them tertiary or granddaughter cysts, by a process of evagination; and on the inner surface of the cysts, whether primary, secondary, or tertiary, the heads or scolices of the immature worm are formed.

The disease is most common between the age of twenty-four and thirty, and the symptoms produced depend upon the organs involved, the size of the tumor, and the inconvenience which results from the pressure and contact upon other structures. The parasite produces no specific symptoms of itself, and its presence might not be detected were it not for the irritation, inflammation, and hyperplasia of the organ which shelters it; but the discomfort is often so slight that the disease remains unrecognised, and is only found out at postmortem. Sometimes the cyst grows rapidly, and its capsule is thinned from pressure necrosis and ruptures, the contents of the sac gets into one or more of the body cavities, and may produce very serious inflammatory disturbances, or an abscess forms and points under the skin, and either breaks or is relieved by an operation.

The first case that has ever been under my notice in Buffalo was brought to me by Dr. Tartaro in April, 1907. The patient was an Italian, in good physical condition, aged thirty-seven; married, and a stone mason by occupation. For a number of months he had noticed a hard, painless swelling in the pit of the stomach, which was gradually getting larger, and was beginning to inconvenience him in his work because of its size. Upon examination, a large globular tumor could be felt, extending deep under the border of the ribs, and particularly to the left side, and filling up practically the whole epigastric and one-half of the inner right hypochondriac region. It was painless, elastic, smooth in outline, and, upon auscultation and palpation, no adventitious sound could be heard and no friction be felt. The man had never had syphilis or malarial fever, and never was a drinking man. The suggestion to thrust a trocar into the tumor and remove some of its contents for microscopial examination was deprecated, on account of the danger attending this simple and seeming trivial undertaking. Cases have been reported where the patient suddenly died after puncture from a profound toxemia, which resulted from the escape of a few drops of the fluid contents of the sac, and in others a milder form of intoxication took place, associated with fever, malaise, an urticarial rash, and other sequelæ. Moreover, one cannot tell what important structures the trocar would pass through in puncturing the cyst wall, since the tumor, as in this instance, was deep and completely covered by bowels and omentum. It would seem, therefore, much safer to make an exploratory incision, and through it a careful examination, and then proceed with such surgical measures as would be indicated, since nothing short of radical surgery can do any good in this disease.

After studying the case from all standpoints, I was forced to concur in the diagnosis of echinococcus cyst of the liver, which Dr. Tartaro had made at an earlier visit. The patient was sent to the German Deaconess's Hospital, and was operated on April 17, 1907. An incision was made a little to the right of the median line, and after getting into the peritoneal cavity the bowels were seen densely adherent all over, and completely covering a large, white, tense, tumor. They were separated with much difficulty, the skin incision was extended freely, the liver was lifted up as much as possible into the wound, and as it was elevated gauze towels were packed deeply underneath the tumor, and about it and the edges of the wound. Some time was occupied in making the gauze packing so complete that the peritoneal cavity and the edges of the incision would be thoroughly protected when the cyst was opened. A large trocar was thrust into the tumor, but nothing came out but a little milky-looking fluid. Then the capsule was freely incised, and at least two quarts of cysts, of varying sizes—from a grain of shot to a large walnut—welled out. After the sac was pretty well emptied, it was irrigated with salt solution through a large glass nozzle, with some force, and pushed to the bottom of the cavity, when the mother cyst or that one lining the capsule began to loosen, and was finally washed away. A small piece of iodoform gauze was then loosely packed into the cavity for drainage; the gauze towels were removed, and the sac around the opening was sewed to the abdominal wall, the wound being closed with through-and-through silkworm gut sutures. After the gauze packs had been removed it was seen that the cyst had sprung from the left lobe of the liver and rested high up on the crura of the diaphragm.

The man rallied nicely, and in a few days was in splendid condition. On the fourth day he had some temperature and developed a slight cough, a friction rub could be heard on the right side, and subsequently all the physical signs and symptoms of pneumonia developed. He ran an evening temperature of 104° and 105°; had considerable cough and bloody expectoration, and became very sick; whereupon Dr. Tartaro gave him the serum treatment, with most satisfactory results. On the eleventh day his temperature was normal; on the fifteenth day the sutures were removed. The wound looked fine, and everywhere it seemed to be perfectly agglutinated. On the seventeenth day he complained of a good deal of pain in the wound, so the dressings were removed, when it was found that the incision had opened and a piece of bowel about four inches long was protruding through the opening. He was taken to the operating room, and, without any anesthetic and without washing the parts, the bowel was gently pushed back; four through-and-through silkworm sutures were passed, and the parts were properly brought together. No complications set in and no pus formed. The sac cavity was occasionally irrigated with salt solution, and he left the hospital in May, 1907; the sinus was still discharging.

I saw him again in July, 1907, when the wound was thoroughly united, and his physical condition was excellent. Perhaps the incomplete union and the subsequent rupture, with bowel protrusion, were due to the delayed and imperfect solidification of the tissues consequent upon the pneumococcus infection, as the sputum revealed the organism in great abundance, and Dr. Tartaro tells me it is in the unmixed or true infections that the serum, which is an Italian product, is most efficacious. It has been used very largely by him, and some of his cases have been reported in the BUFFALO MEDICAL JOURNAL, in an article read in March, 1906, by him before the Buffalo Academy of Medicine.

493 DELAWARE AVENUE.

---

JUST AS GOOD AS EVER, TOO.—An old physician was noted for his brusque manner and old-fashioned methods. A lady called him in to treat her baby, who was slightly ailing. The doctor prescribed castor oil.

"But, doctor," protested the young mother, "castor oil is such an old-fashioned remedy."

"Madam," replied the doctor, "babies are old-fashioned things."
—*London Opinion.*

